# Effect of *Bifidobacterium longum* subsp. *infantis* YLGB-1496 on common diseases in pediatrics: a randomized, blinded, placebo-controlled trial

**DOI:** 10.3389/fnut.2025.1585504

**Published:** 2025-06-27

**Authors:** Xi Zhang, Ke Chen, Hanglian Lan, Haixia Chen, Hua Chen, Ping Yang, Nianyang He, Weilian Hung, Zaozhong Zeng, Changqi Liu

**Affiliations:** ^1^Chengdu Women's and Children's Central Hospital, School of Medicine, University of Electronic Science and Technology of China, Chengdu, China; ^2^Department of Nutrition, Chengdu Women's and Children's Central Hospital, School of Medicine, University of Electronic Science and Technology of China, Chengdu, China; ^3^National Center of Technology Innovation for Dairy, Hohhot, China; ^4^Baoxing Center for Disease Control and Prevention, Yaan, China; ^5^Department of Child Health Care, Xindu Maternal and Child Health Care Hospital, Chengdu, China; ^6^School of Exercise and Nutritional Sciences, San Diego State University, San Diego, CA, United States

**Keywords:** probiotics, short chain fatty acids, children, gut microbiota, upper respiratory tract infection

## Abstract

**Introduction:**

Respiratory, gastrointestinal, and allergic diseases can significantly impact both the physical and mental health of children, affecting their overall quality of life. This study aimed to evaluate the preventive effects and safety of *Bifidobacterium longum* subsp. *infantis* YLGB-1496 in relation to respiratory, gastrointestinal, and allergic diseases in children.

**Methods:**

Eligible healthy children were randomly assigned to either an intervention group (IG, *n* = 50), which received the probiotic, or a control group (CG, *n* = 50), which received a placebo, for a duration of 3 months. The primary outcome was the morbidity of upper respiratory tract infections (URTIs). Gut microbiota profiles were assessed via fecal 16S rRNA sequencing. Fecal immune biomarkers, including cytokines, immunoglobulins, and short-chain fatty acids (SCFAs), were measured to evaluate immune and metabolic responses.

**Results:**

The morbidity of URTIs over the 3-month intervention and follow-up period was significantly lower in the IG than in the CG. The incidence of upper respiratory tract infections (URTIs) over the 3-month intervention and follow-up period was significantly lower in the IG than in the CG, based on intention-to-treat (ITT) analysis [34.0% (17/50) vs. 58.0% (29/50), χ^2^ = 5.797, *p* = 0.016]. Per protocol (PP) analyses yielded similar results [36.2% (17/47) vs. 60.4% (29/48), χ^2^ = 5.59, *p* = 0.018]. YLGB-1496 supplementation significantly increased the relative abundance of *Bifidobacterium bifidum, Bifidobacterium kashiwanohense* PV2, and *Bifidobacterium longum*, while reducing *Bacteroides thetaiotaomicron* levels in the IG compared to the CG (*p* < 0.05). Additionally, YLGB-1496 reduced fecal levels of pro-inflammatory factors (IL-1β and IFNγ) levels, and increased levels of immunoglobulin (IgA, IgG, and IgM) and SCFAs (including butyric acid and total SCFAs).

**Conclusion:**

Daily administration of YLGB-1496 at a dosage of 1.5 × 10^10^ CFU for 3 months significantly reduced the episodes of cough, fever, dry stool (defined as Bristol stool scale type 1–3), and eczematous changes of the skin. It also decreased the morbidity of URTIs, bronchopneumonia, and eczema, while beneficially modulating gut microbiome composition and immune function without any adverse effects.

**Clinical trial registration:**

https://www.isrctn.com/ISRCTN12824613, identifier: ISRCTN12824613.

## 1 Introduction

The World Health Organization (WHO) estimated that in 2019, ~5.3 million children under the age of five died globally from various causes. Among these fatalities, infectious diseases accounted for 49.2%, with respiratory and gastrointestinal diseases being the leading contributors, responsible for 13.9 and 9.1% of deaths, respectively (1). Concurrently, the incidence of allergic diseases has risen substantially over the past three decades, posing a major public health challenge in many developed countries (2), including China (3). According to the WHO asthma fact sheet, allergic diseases impact ~433 million people worldwide (4). Preventing respiratory, gastrointestinal, and allergic diseases is therefore crucial for the health and development of infants and young children.

Breastfeeding is widely recognized as a cost-effective intervention (5) to reduce childhood mortality and prevent common infections. Breast milk contains a variety of anti-infective, anti-inflammatory, and immunomodulatory components that strengthen the child's immune system (6, 7). It also provides beneficial bacteria that support the colonization of a healthy gut microbiota (6). Similarly, probiotics can help establish and maintain the intestinal mucosal barrier, facilitate immune development, and protect against intestinal infections (8, 9). A growing body of research suggests that probiotics can prevent and alleviate common childhood illnesses by modulating gut microbiota and immune responses (10–13), including findings from our own previous studies (14–20). As a result, probiotic supplements have gained traction as adjuncts for the treatment and prevention of these diseases, with hundreds of products now available on the market. These products differ widely in their excipients, quantities, microbial strains, and clinical efficacy (21). Importantly, the effects of probiotics are highly strain specificity (22, 23).

Strains isolated from human breast milk are considered promising candidates for probiotic development, and numerous studies have focused on characterizing these strains for infant health applications (6). *Bifidobacterium longum* subsp. *infantis* YLGB-1496 is a proprietary strain isolated from human breast milk and registered with the China Center for Type Culture Collection (CCTCC No. M2011122). Animal studies and *in vitro* studies have shown that YLGB-1496 exhibits strong oxygen free radical scavenging activities, bactericidal and bacteriostatic effects against *Escherichia coli*, and immune-regulating capabilities (24–26).

To the best of our knowledge, no study has yet investigated whether YLGB-1496 can enhance immune function and reduce the incidence of respiratory, gastrointestinal, and allergic diseases in children. To address this gap, we conducted a randomized, placebo-controlled, double-blind trial in several communities on the outskirts of Chengdu City. This study aimed to evaluate whether daily administration of YLGB-1496 could reduce the incidence of these common diseases in children, while also positively influencing gut microbiota and intestinal immunity.

## 2 Materials and methods

### 2.1 Study design and participants

This study was a prospective, parallel, multi-center, double-blinded, randomized, placebo-controlled clinical trial conducted between August 2024 and February 2025. The study enrolled healthy children of both sex aged 0–3 years from three sites in Sichuan Province, China: Chengdu Women and Children's Center Hospital, Baoxing Center for Disease Control and Prevention, and Xindu Maternal and Child Health Care Hospital.

The inclusion criteria were: (1) healthy children aged 0–3 years who were not breastfed, born at 37–42 weeks of gestation, and with a birth weight between 2,500 and 4,000 g; (2) parents and/or guardians agreeing to collect feces sample throughout the study period; (3) no diagnosed allergic diseases (including but not limited to eczema, food allergies, asthma, allergic colitis, allergic rhinitis, and hay fever) at enrollment; (4) no use of other probiotics during the trial; (5) written informed consent provided by parents or guardians.

The exclusion criteria were: (1) history of asphyxia or neonatal intensive care unit (NICU) admission at birth; (2) congenital defects or anomalies; (3) obstetric risk factors during pregnancy such as gestational hypertension syndrome, eclampsia, gestational diabetes, cholestasis of pregnancy; (4) use of antibiotics within 2 weeks prior to enrollment; (5) definitive diseases that impact growth and development within the month prior to enrollment (e.g., pneumonia, severe diarrhea or constipation, malnutrition, gastrointestinal surgery, epilepsy, cerebral palsy, intellectual disabilities, and confirmed genetic metabolic disorders, chromosomal abnormalities, genetic conditions); (6) use of experimental medications or participating in another clinical trial before screening; (7) prior use of probiotic products containing YLGB-1496 within 1 month of enrollment; (8) use of immunosuppressive drugs (e.g., glucocorticoids, immunosuppressants); (9) known allergies to any components of the probiotic products used in the study; (10) other reasons deemed inappropriate for participation in the clinical trial by the researchers, such as factors affecting efficacy evaluation or poor compliance.

Withdrawal criteria: (1) incorrect inclusion and misdiagnosis; (2) lack of clinical records for assessment; (3) allergies to ingredients in the probiotic products during the trial; (4) oral probiotics cannot be taken by mouth during the intervention; (5) clinical deteriorated requiring admission to the pediatric intensive care unit (PICU).

The enrollment and research plan were reviewed and approved by the Ethics Committee of the Baoxing Center for Disease Control and Prevention [Ethical Approval No.: Scientific Ethics Review 2024 (01)]. Written informed consent was obtained from the parents or legal guardians of each child. The study adhered to the ethical guidelines outlined by the World Medical Association (Declaration of Helsinki) and was registered with the International Standard Randomized Controlled Trial Number (ISRCTN) registry (Registration No: ISRCTN12824613; https://www.isrctn.com/ ISRCTN12824613). Project planning began in January 2024, with official launch and registration in July 2024 (Registration date: July 22, 2024). Participant recruitment started in August 2024 and was completed in February 2025.

This study was supported by the National Center of Technology Innovation for Dairy (No. 2022-KYGG-6).

### 2.2 Randomization and blinding

Randomization was conducted by a biostatistician who was not involved in the implementation of the trial. Using the RAND function in Excel, random numbers were generated to assign eligible participants to either the probiotic intervention group or the placebo control group. Participants who met the inclusion criteria were sequentially numbered and randomly allocated, resulting in 50 children per group. The probiotic and placebo sachets were indistinguishable in appearance, taste, and smell. Both were packaged in identical sachets with matching labels, differing only by the subject-specific randomization number. The parents/guardians, clinicians, laboratory personnel, data manager, and statistician were blinded to group assignments until completion of the data analysis.

### 2.3 Sample size

Sample size estimation was informed by a previous study (27) that investigated the preventive effects of probiotics on upper respiratory tract infections (URTIs) symptoms in children. In that study, the frequency of URTI episodes during the 3rd month of probiotic supplementation was 0.24 times/person in the intervention group [*n* = 38 for per protocol (PP) analysis, *n* = 50 for intention-to-treat (ITT) analysis] vs. 0.73 times/person in the placebo group (*n* = 33 for PP analysis, *n* = 50 for ITT analysis), with a significant difference (*p* < 0.001). Based on these findings, We enrolled 50 participants in each group to ensure that the intention-to-treat (ITT) analysis would have adequate power to detect a statistically significant difference in URTI incidence between groups.

### 2.4 Intervention

All eligible children were randomly assigned to either the intervention group (IG) or the control group (CG). Children in the IG received a daily oral probiotic sachet (Wecare Probiotics Co., Ltd., production No: SC10632050900407). Each sachet contained YLGB-1496 and maltodextrin, with a total viable count of 1.5 × 10^10^ CFU per sachet. The sachet could be taken directly or mixed with warm water (below 45°C), milk, rice porridge, or other liquid foods. The intervention lasted for 90 consecutive days, starting from the first day of study enrollment. Children in the CG received a placebo sachet containing only maltodextrin. The probiotic and placebo were indistinguishable in appearance, taste, and smell, and were packaged in identical sachets with matching labels, differing only by the subject-specific randomization number. If a child vomited within 30 min of taking the sachet, an additional dose could be administered (limited to one extra sachet within a 4-h period). All dosing and re-dosing were documented in a case report form (CRF) by the treating physicians. During the intervention period, the subjects had monthly on-site follow-up visits. Parents and caregivers were able to communicate and consulted with the study team at any time for questions or concerns. Any illnesses during the trial were managed by pediatrician according to standard clinical guidelines. In cases where probiotics and oral antibiotics were prescribed concurrently, a minimum interval of 3 h was maintained to avoid interference.

### 2.5 Data collection

Following enrollment, the study team conducted assessments, documented data in case report forms (CRFs), and collected biological samples according to the study protocol. The primary outcome was the frequency of URTIs (28) during the 3-month period. Diagnosis, treatment, and clinical management of URTIs were determined according to the relevant guidelines of the Chinese Medical Association (adding reference here). Secondary outcomes included the incidence of other respiratory, gastrointestinal, and allergic diseases, as well as stool-based biochemical indicators, including pro-inflammatory/inflammatory markers: tumor necrosis factor-α (TNF-α), interleukin-6 (IL-6), IL-1β, interferon-γ (IFN-γ), and calprotectin; anti-inflammatory factors: IL-10, transforming growth factor-β (TGF-β), immunoglobulin A (IgA), IgG, and IgM; allergy-related cytokines: IL-4 and IL-5; short-chain fatty acids (SCFAs): acetic acid, propionic acid, butyric acid, and total SCFAs; and gut microbiome profiles over the 3-month intervention period. Clinicians used the CRFs to document the duration, frequency, therapeutic drugs, and related symptoms and signs of respiratory, gastrointestinal, and allergic diseases over the course of the 3-month study period.

### 2.6 Sample collection and biochemical analysis

Fecal samples were collected at baseline (week 0) and at the end of the intervention (week 12) by parents/guardians using fecal collection tubes containing RNAlater™ solution and glass beads. Samples were sealed and stored at −80°C until analyses. Biochemical indicators in stool were determined using human protein-specific enzyme-linked immunosorbent assay (ELISA) kits (Shanghai Enzyme Linked Biotechnology Co., Ltd., Shanghai, China), following the manufacturer's instructions. Protein concentrations were calculated based on standard curves generated using protein standards and reported in arbitrary units per milliliter.

For SCFAs analysis, fecal aliquots were weighed, acidified with 2 N hydrochloric acid, and stored at −20°C. Before analysis, samples were thawed and centrifuged at 13,000 *g* for 10 min (29). Quantification of SCFAs (acetic acid, propionic acid, butyric acid, and total SCFAs) was performed via gas chromatography (Hewlett–Packard 5890 A Series II, 180 cm × 4 mm i.d. glass column packed with 10% SP-1200/1% HVFA H_3_PO_4_ on 80/100 mesh Chromosorb WAW, Supelco, Inc., Bellefonte, PA). SCFA concentrations were normalized to fecal dry matter weight, as described in a previous study (29).

### 2.7 Microbiota analyses

Genomic DNA (30) was extracted from fecal samples using the Cetyltrimethylammonium Bromide/Sodium Dodecyl Sulfate (CTAB/SDS) method with the QIAamp Fast DNA Fecal Mini Kit (Qiagen, Valencia, California, USA), following the manufacturer's protocol. The V3-V4 region of the bacterial 16S rRNA gene was amplified using the TransGen AP221-02 Kit (TransGen, Beijing, China) with the 16S V34: 341F-806R Polymerase Chain Reaction (PCR) primers. For sequence analysis, Uparse software (v7.0.1001) and Quantitative Insights Into Microbial Ecology (QIIME, version 1.9.1) software were employed. Sequences exhibiting ≥97% similarity were grouped into the same operational taxonomic units (OTUs). Representative sequences from each OTU were selected and taxonomically annotated using the Ribosomal Database Project (RDP) classifier. Alpha diversity (within sample diversity) was assessed using Shannon, Simpson, Chao1, and Abundance-based Coverage Estimator (ACE) indices. Beta diversity (between sample diversity) was evaluated using principal coordinate analysis (PCoA) based on Bray–Curtis distance. The differential abundance of taxa between groups was assessed using the Kruskal–Wallis test.

### 2.8 Statistical analyses

Primary outcome analysis was performed using both ITT principles and PP dataset. The Kolmogorov–Smirnov goodness-of-fit test was conducted to assess the normality of data distributions. Descriptive statistics were reported as mean ± standard deviation (SD) or median (P25, P75) for continuous variables, and as counts and percentages for categorical variables. When appropriate, data were log-transformed to normalize distributions.

For comparisons between groups, the following statistical tests were applied: for normally distributed variables with equal variance, two-independent-sample Student's *t*-test was used; for non-normally distributed data, the Mann–Whitney *U*-test was used; for categorical variables, the chi-square (χ^2^) test was applied. Negative binomial regression was used to evaluate the relative risk and 95% confidence interval for the duration and frequency of symptoms associated with respiratory, gastrointestinal, and allergic diseases following probiotic intervention.

A binary multivariate logistic regression was carried out to assess the impact of the intervention on the incidence of respiratory, gastrointestinal, and allergic diseases throughout the study period. All analyses were conducted using IBM SPSS Statistics (version 29.0.2 for Mac).

## 3 Results

### 3.1 Baseline characteristics

**Figure 1:**
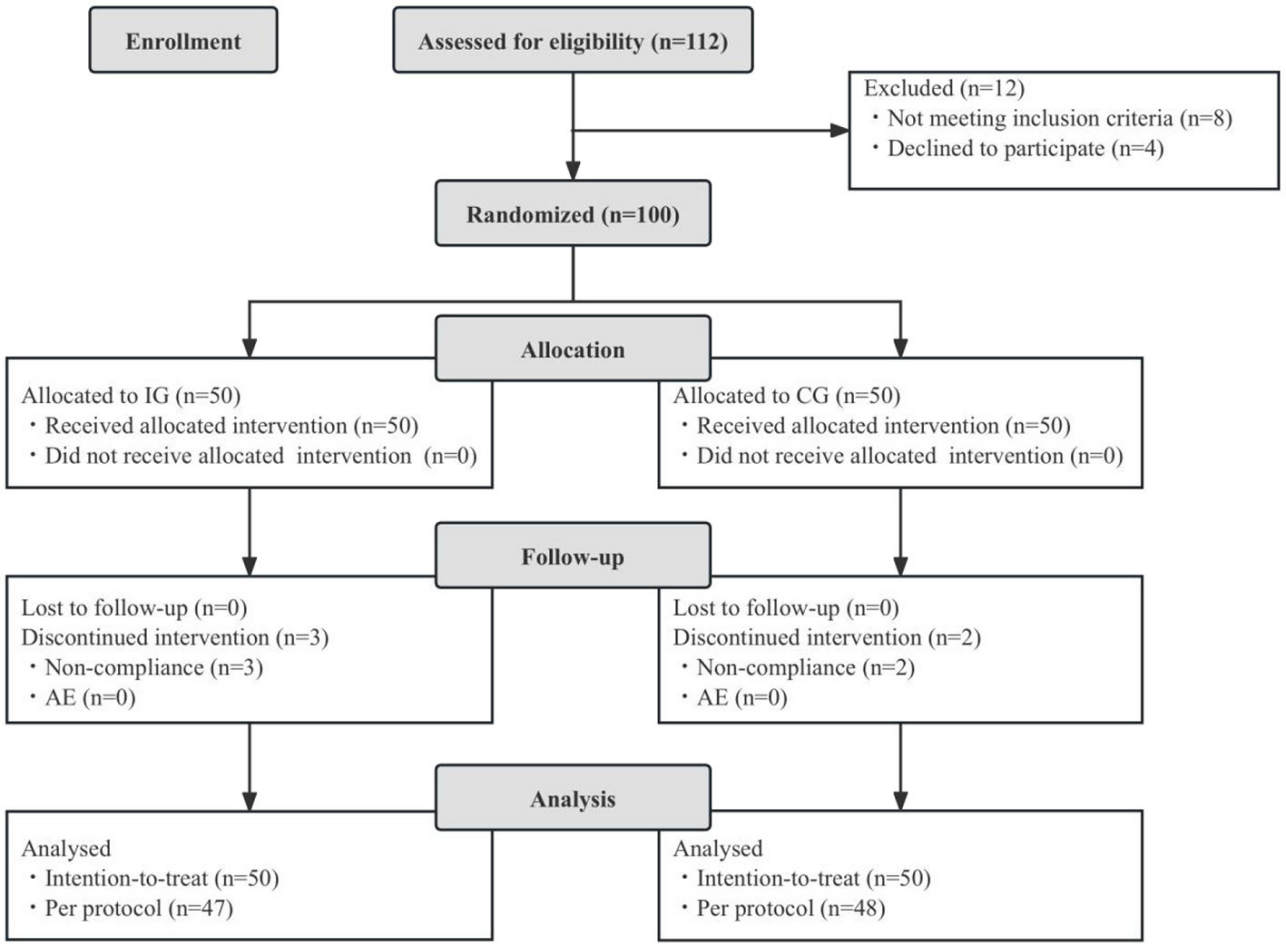
Flowchart of subject enrollment and study progress. IG, intervention group; CG, control group; AE, adverse events.

A total of 100 eligible children were enrolled in the study. All participants completed the full intervention procedures and provided clinical and demographic data. All enrolled children were randomly assigned to either the intervention group (IG, *n* = 50) or the control group (CG, *n* = 50), and were included in the intention-to-treat (ITT) analysis. There were no losses to follow-up, and all participants completed their case report forms (CRFs). However, five children were excluded from the per-protocol (PP) analysis due to significant protocol deviations. Consequently, the PP dataset included 95 children: 47 in the IG and 48 in the CG. Throughout the study, no adverse events related to the study product were reported. Figure 1 presents the flowchart outlining participant recruitment, allocation, follow-up, and analysis. At baseline, there were no significant differences between the two groups in terms of sex distribution, age, parental education level, monthly per capita household income, registered residence, mode of delivery, or number of household residents (*p* > 0.05, Table 1).

**Table 1:** Basic clinical and demographic information of the two groups [means ± SD].

**Items**	**IG (*n* = 50)**	**CG (*n* = 50)**	***t/χ^2^* value**	***p*-Value**
Sex, [boy *n* (%)]^*^	27 (54.0)	23 (46.0)	0.64	0.423
Age (months)^*^	14.5 ± 10.3	16.9 ± 10.6	1.148	0.126
Birth weight (kg)^*^	3.16 ± 0.5	3.12 ± 0.6	0.362	0.641
Parental education level^a*^			1.08	0.780
Junior high school and below	9	6		
Senior high school/technical secondary school	13	12		
Junior college/vocational college	16	20		
Bachelor's degree or above	12	12		
Household per capita income^*^			7.98	0.092
<5,000 RMB¥	33	41		
5,001–6,000 RMB¥	10	4		
>6,001 RMB¥	7	3		
Registered residence [urban, *n* (%)]^*^	29 (58.0)	33 (66.0)	0.679	0.410
Delivery mode [vaginal, *n* (%)]^*^	19 (38.0)	21 (42.0)	0.167	0.683
Number of household residents [*n* < 4, n (%)]^*^	12 (24.0)	18 (36.0)	1.714	0.190

^a^Selecting the highest educated level of parents as the statistical data. ^*^No significant difference between the IG and the CG (*p* > 0.05); SD, standard deviation; IG, intervention group; CG, control group.

### 3.2 Effect of probiotic intervention on the primary outcome

The incidence of URTIs over the 3-month intervention and follow-up period was significantly lower in the IG compared to the CG [34.0% (17/50) vs. 58.0% (29/50), respectively, χ^2^ = 5.797, *p* = 0.016] according to the ITT analysis.

The PP analyses yielded consistent results, with significantly lower URTI incidences in the IG than in the CG [36.2% (17/47) vs. 60.4% (29/48), respectively, χ^2^ = 5.59, *p* = 0.018]. These findings indicate a statistically significant reduction in URTI incidence with probiotic supplementation, consistent across both ITT and PP analyses.

#### 3.2.1 Effect of probiotic intervention on symptom duration of respiratory, gastrointestinal, and allergic diseases

During the 3-month study period, the duration of common respiratory, gastrointestinal, and allergic symptoms, expressed as incidence rate per 100 intervention days, is summarized in Table 2. Children in the IG experienced significantly shorter durations of common symptoms such as cough, fever, dry stool (defined as Bristol stool scale type 1–3), and eczematous changes of the skin compared to those in the CG (*p* < 0.05 for all). After adjusting for potential confounders including gender, age in months, parental education levels, monthly household income per capita, registered residence, mode of delivery, and number of household residents, negative binomial regression analyses revealed that probiotic intervention effectively reduced the risk of fever (*B* = 1.064, 95% CI = 0.422–1.705), dry stool (*B* = 0.863, 95% CI = 0.339–1.387), eczematous changes of the skin (*B* = 1.508, 95% CI = 1.014–2.002; *p* < 0.05, respectively; Supplementary Table 1).

**Table 2:** Comparison of the days of common symptoms in infants and young children during the intervention period.

**Common symptoms**	**CG (*****N*** = **50)**		**IG (*****N*** = **50)**		***p-*value**
	**Total occurrence days**	**Incident rate per 100 intervention days (%)^†^**	**Total occurrence days**	**Incident rate per 100 intervention days (%)^†^**	
Cough^*^	190	4.22	99	2.2	< 0.001
Runny nose	171	3.8	136	3.02	0.133
Stuffy nose	120	2.67	83	1.84	0.066
Fever^*^	99	2.2	40	0.89	< 0.001
Loose stools	97	2.16	120	2.67	0.413
Increased frequency of bowel movements	105	2.33	105	2.33	0.921
Colic of the intestines	105	2.33	102	2.27	0.979
Dry stool^*^	346	7.69	157	3.49	0.012
Choking	45	1	58	1.29	0.387
Increased belching/bloating/anal gas	87	1.93	89	1.98	0.915
Retching/vomiting	35	0.78	52	1.16	0.244
Stool with milk flaps/food residues/sour odor	183	4.07	194	4.31	0.867
Reflux	58	1.29	64	1.42	0.393
Decreased appetite	181	4.02	135	3	0.285
Dysphoria	225	5	151	3.36	0.659
Refusal to eat	83	1.84	145	3.22	0.065
Eczematous changes of the skin^*^	578	12.84	134	2.98	0.002
Erythematous changes in the skin	12	0.27	9	0.2	0.958
Skin wheal-like changes	10	0.22	7	0.16	0.552

^†^A total of 9,000 study days (calculated method: 180 days × 50 people). Symptom incidence per 100 intervention days = duration of a symptom/total intervention days × 100%.

^*^Mann–Whitney *U*-test for two independent samples, *p*-values < 0.01. Dry stool refers to stool Bristol score type 1–3 stool.

IG, intervention group; CG, control group.

#### 3.2.2 Effect of probiotic intervention on the morbidity of respiratory, gastrointestinal, and allergic diseases

Morbidity data were collected over a median of 180 days (interquartile range 169–187 days) for both groups. Table 3 summarizes the incidences and the risk ratios (RR) for respiratory, gastrointestinal, and allergic diseases during the study period. Compared to the CG, the IG showed a reduced risk of new URTI (34.0 vs. 58.0%, RR = 0.586, 95% CI = 0.373–0.922), bronchopneumonia (6.0 vs. 22.0%, RR = 0.273, 95% CI = 0.081–0.919), and eczema (32.0 vs. 56.0%, RR = 0.647, 95% CI = 0.449–0.933).

**Table 3:** Morbidity of respiratory, gastrointestinal, and allergic diseases in infants and young children during the study period.

**Episodes**	**IG (*****N*** = **50)**		**CG (*****N*** = **50)**		**RR**	**95% CI**
	**Numbers of episodes**	**Incidence (%)**	**Numbers of episodes**	**Incidence (%)**		
URTIs^*^	17	34.0	29	58.0	0.586	0.373–0.922
Acute tracheal/bronchitis	4	8.0	9	18.0	0.444	0.146–1.349
Bronchopneumonia^*^	3	6.0	11	22.0	0.273	0.081–0.919
Infantile diarrheal disease	11	22.0	14	28.0	0.786	0.396–1.560
Functional constipation in infants and young children	2	4.0	3	6.0	0.667	0.116–3.820
Infantile intestinal spasms	3	6.0	1	2.0	3.000	0.323–27.871
Functional dyspepsia syndrome in children	18	36.0	23	46.0	0.783	0.486–1.260
Eczema^*^	16	32.0	28	56.0	0.647	0.449–0.933

^*^Significant difference of the incidence between the two groups (*p* < 0.05). URTIs, upper respiratory tract infection; RR, risk ratio; 95% CI, 95% confidence interval for risk ratio; IG, intervention group; CG, control group.

### 3.3 Effect of probiotic intervention on growth parameters

There were no significant differences in weight, length, or head circumference between the two groups at baseline (*p* > 0.05). By the end of the study, all three growth parameters had increased relative to the baseline levels; however, the differences between the two groups remained statistically non-significant (*p* > 0.05; Supplementary Table 2).

### 3.4 Effect of probiotic intervention on fecal gut microbiota

#### 3.4.1 Effect on alpha diversity

**Figure 2:**
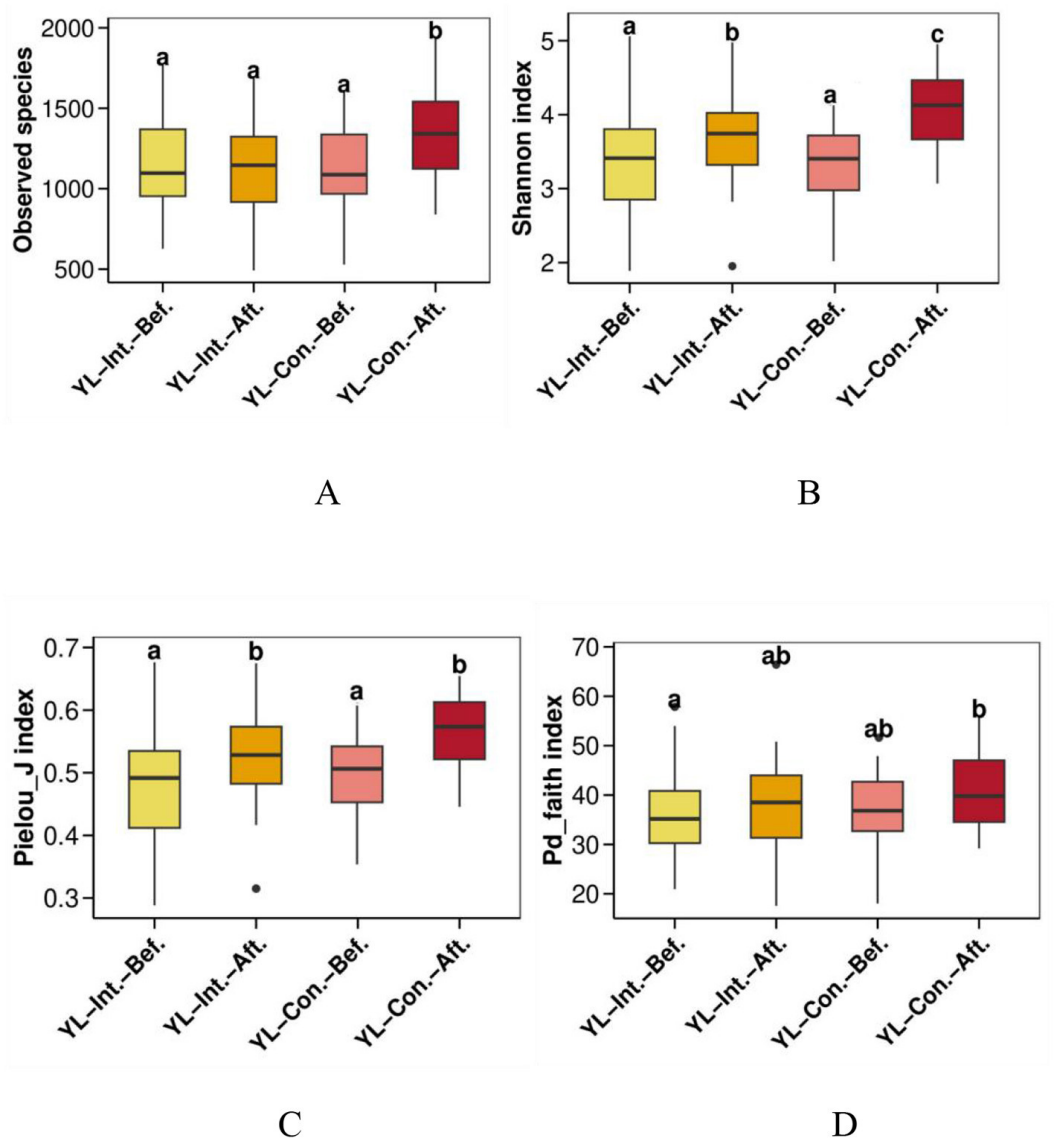
Effect of intervention on alpha diversity indices of the gut microbiota. **(A)** Observed species richness index; **(B)** Shannon diversity index; **(C)** Pielou_j evenness index; **(D)** Pd_faith evolutionary diversity index. abc, bars sharing the same letters indicate no significant difference between groups (*p* > 0.05); bars with different letters indicate statistically significant differences (*p* < 0.05). YL-Int.-Bef., intervention group before intervention; YL-Int.-Aft., intervention group after intervention; YL-Con.-Bef., control group before intervention; YL-Con.-Aft., control group after intervention.

Alpha diversity was assessed to evaluate the richness and evenness of microbial community within each sample (Figure 2). QIIME software was used to calculate four alpha diversity indices: observed species (richness), Shannon (diversity), Pielou_J (evenness), and Pd_faith (evolutionary diversity). At baseline, there were no significant differences in any of the four alpha diversity indices between the two groups (*p* > 0.05). After the intervention, both Shannon and Pielou_J indices increased significantly in both groups (Figures 2B, C, *p* < 0.05), while the Pd_faith index remained unchanged (Figure 2D, *p* > 0.05). The observed species index remained stable in the IG (Figure 2A, *p* > 0.05) but increased in the CG (Figure 2A, *p* < 0.05). After the intervention, the observed species and Shannon indices were significantly lower in the IG compared to the CG (Figures 2A, B, *p* < 0.05).

#### 3.4.2 Effect of probiotic intervention on beta diversity of fecal gut microbiota

##### 3.4.2.1 Beta diversity distance visualization

**Figure 3:**
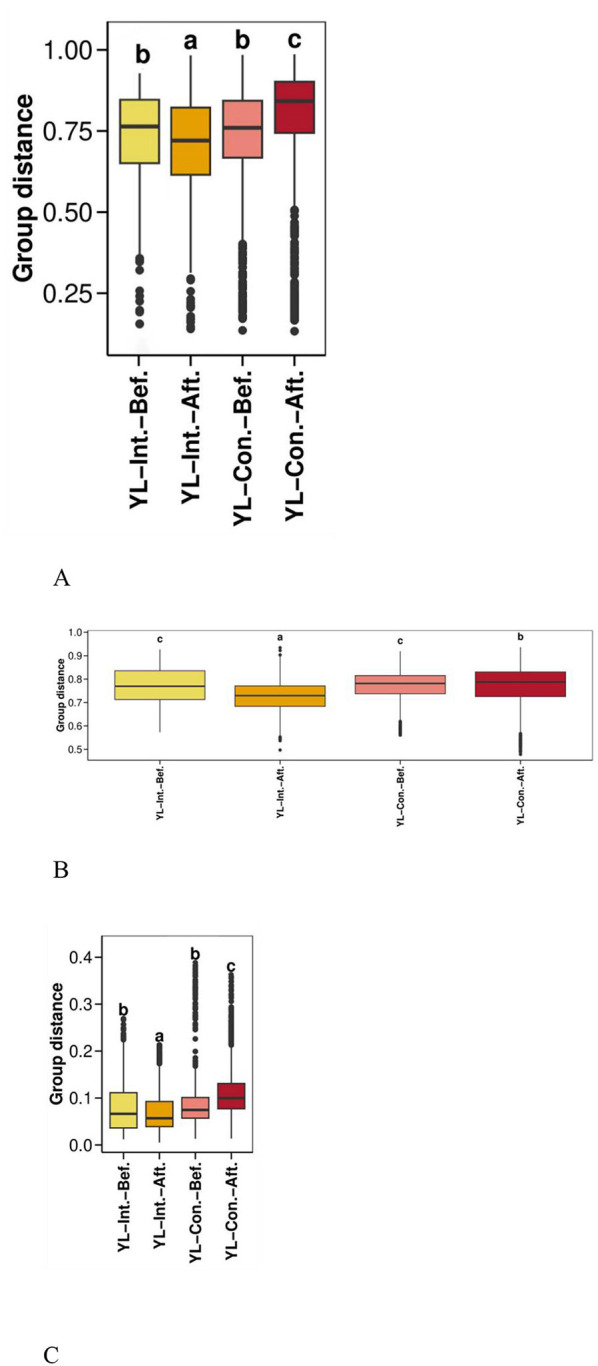
Effect of probiotic intervention on beta diversity distances. Boxplots were generated using distances calculated by Bray Curtis **(A)**, weighted UniFrac **(B)**, and unweighted UniFrac **(C)** to compare the distribution of microbial community dissimilarities within and between groups. YL-Int.-Bef., intervention group before intervention; YL-Int.-Aft., intervention group after intervention; YL-Con.-Bef., control group before intervention; YL-Con.-Aft., control group after intervention. Bars sharing the same letters indicate no significant difference between groups (*p* > 0.05); bars with different letters indicate statistically significant differences (*p* < 0.05).

Boxplots were generated using Bray–Curtis, weighted UniFrac, and unweighted UniFrac distances to visualize beta diversity and compare microbial community dissimilarities within and between sample groups (Figures 3A–C). Before the intervention, no significant differences in beta diversity were observed between the two groups across all three distance metrics (*p* > 0.05). However, after the intervention, all beta diversity distances significantly decreased in the IG (*p* < 0.05), indicating increased similarity within the group's microbial communities. Conversely, the distances significantly increased in the CG (*p* < 0.05), suggesting increased microbial variability over time. In other words, the microbial community dissimilarity in the CG was significantly higher than that in the IG across all metrics (*p* < 0.05).

##### 3.4.2.2 PCA analysis of beta diversity

**Figure 4:**
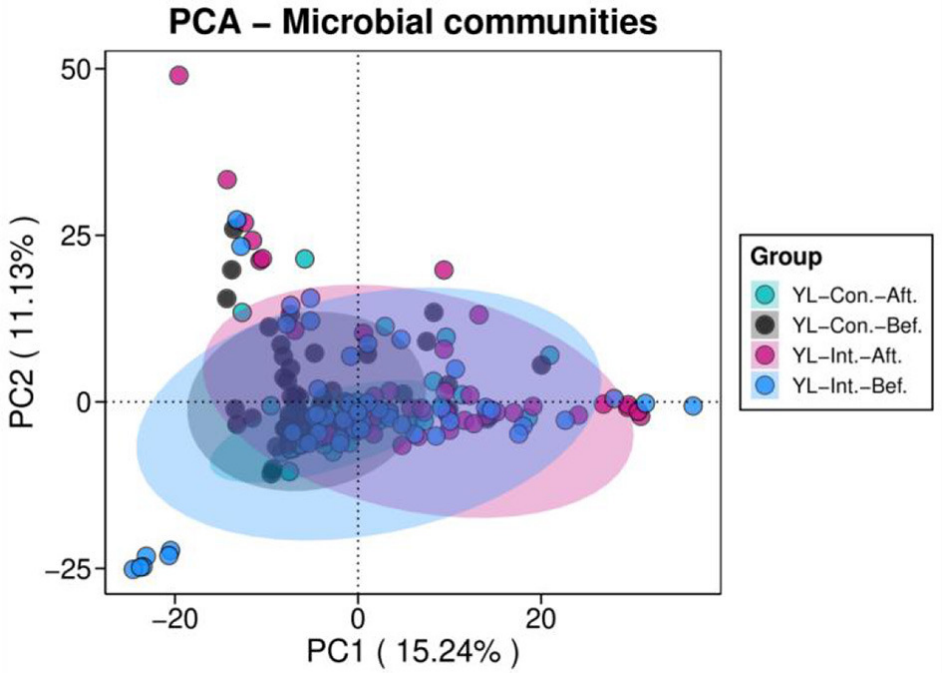
Principal component analyses (PCA) of beta diversity in the intervention and control groups before and after intervention. YL-Int.-Bef., intervention group before intervention; YL-Int.-Aft., intervention group after intervention; YL-Con.-Bef., control group before intervention; YL-Con.-Aft., control group after intervention.

Principal component analysis (PCA) of beta diversity was performed to visualize differences in microbial community structure before and after the intervention (Figure 4). The first principal component (PC1) explained 15.24% of the total variance, while PC2 accounted for 11.13%. Before the intervention, samples from the IG and the CG were closely clustered, indicating similar microbial compositions. However, after the intervention, clear spatial separation was observed between the two groups, suggesting a shift in gut microbiota structure in response to the probiotic treatment (Figure 4).

##### 3.4.2.3 PCoA analysis of beta diversity

**Figure 5:**
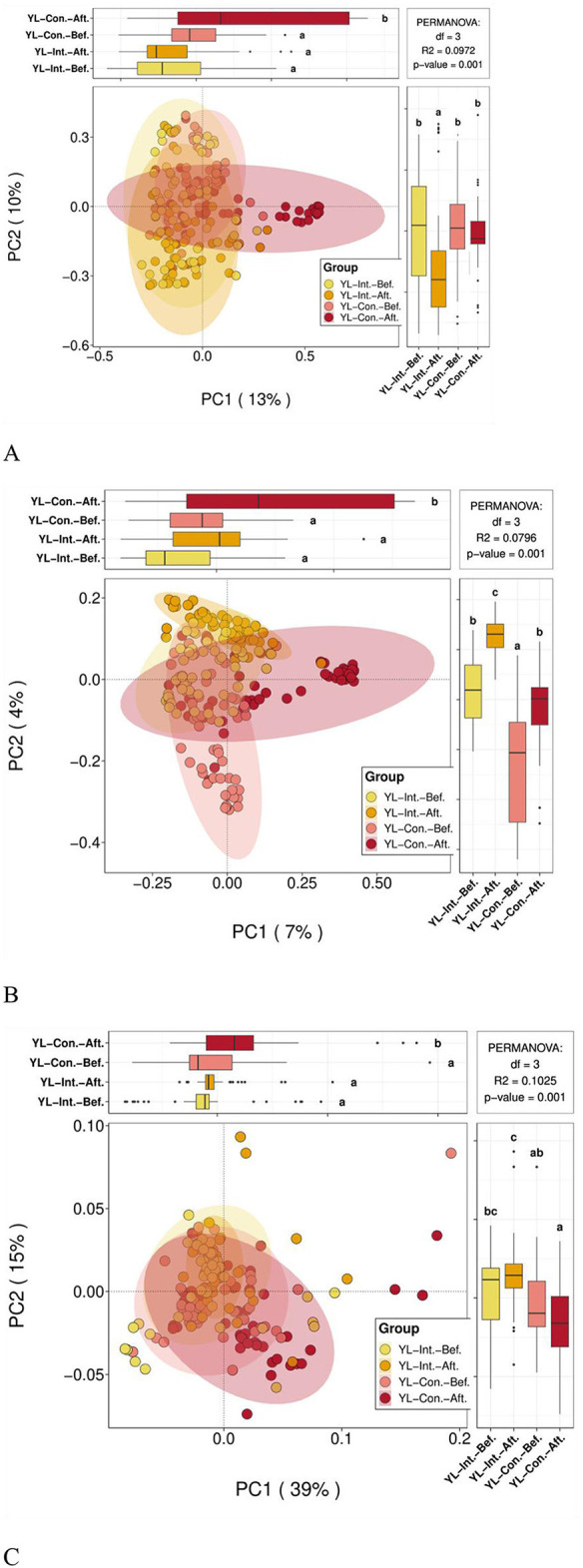
PCoA analysis of beta diversity between groups before and after the intervention. YL-Int.-Bef., intervention group before intervention; YL-Int.-Aft., intervention group after intervention; YL-Con.-Bef., control group before intervention; YL-Con.-Aft., control group after intervention. **(A)** PCoA plots based on Bray-Curtis distance; **(B)** Weighted Unifrac distance; and **(C)** Unweighted Unifrac distance. ^a − c^When comparing groups pairwise, different letters indicate a statistical difference between groups (*p* < 0.05); while the same letters indicate no statistical difference between groups (*p* > 0.05).

PCoA plots based on Bray–Curtis, weighted Unifrac, and unweighted Unifrac distances are presented in Figures 5A–C. Based on the Bray–Curtis distance (Figure 5A), the first principal coordinate (PC1) explained 13% of the total variance, and PC2 accounted for 10%. At baseline, no significant differences in PC1 and PC2 components were observed between the IG and the CG (*p* > 0.05). However, post-intervention, significant differences emerged between the two groups (*p* < 0.05). Specifically, the PC1 scores significantly increased in the CG (*p* < 0.05) but remained unchanged in the IG (*p* < 0.05). The PC2 scores significantly decreased in the IG (*p* < 0.05) but remained stable in the CG (*p* > 0.05). For the weighted Unifrac distances (Figure 5B), PC1 and PC2 explained 7 and 4% of the variance, respectively. After the intervention, PC1 scores increased significantly in the CG (*p* < 0.05), with no significant change in the IG (*p* > 0.05). PC2 scores increased significantly in both groups (*p* < 0.05), with a greater increase observed in the IG (*p* < 0.05). Using the unweighted Unifrac distances (Figure 5C), PC1 and PC2 explained 39 and 15% of the variance, respectively. Prior to the intervention, no significant differences were observed between groups (*p* > 0.05). After the intervention, PC1 scores increased significantly in the CG (*p* < 0.05), but remained unchanged in the IG (*p* > 0.05). PC2 scores showed no significant increase within either group individually (*p* > 0.05), but the post-intervention value was significantly higher in the IG compared to the CG (*p* < 0.05).

##### 3.4.2.4 Comparison of top 10 species-level relative abundance differences between groups

**Figure 6:**
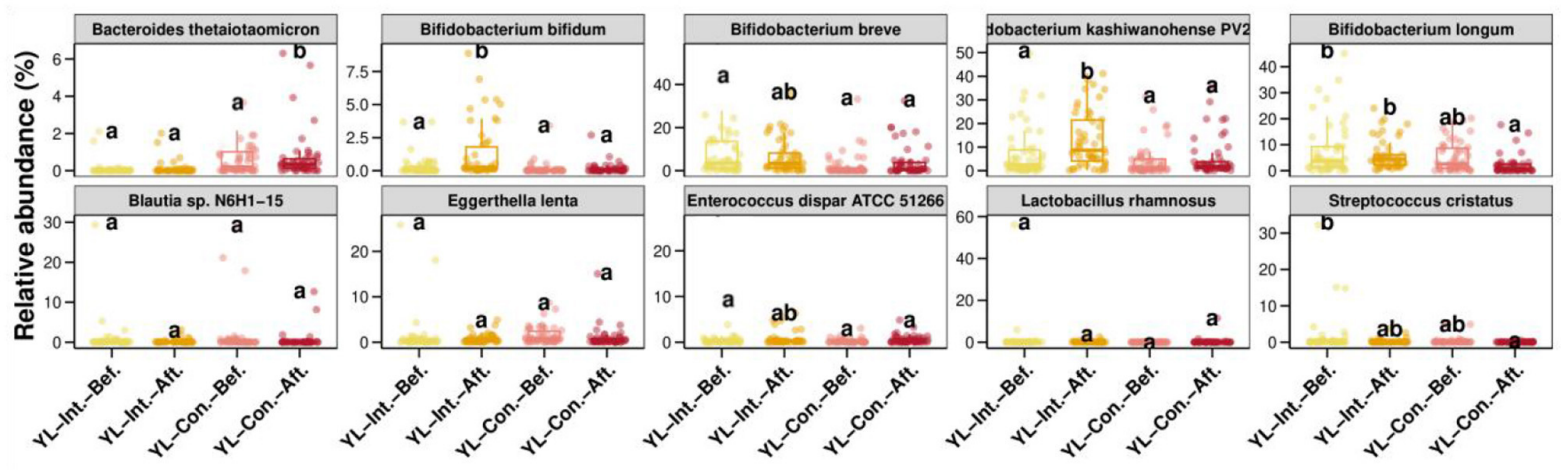
Comparison of the top 10 species-level relative abundance differences between groups. Int.-Bef.-Treatment, intervention group before intervention; Int.-Aft.-Treatment, intervention group after intervention; Con.-Bef-Treatment, control group before intervention; Con.-Aft-Treatment, control group after intervention. Bars sharing the same letters indicate no significant difference between groups (*p* > 0.05); bars with different letters indicate statistically significant differences (*p* < 0.05).

The comparison of the top 10 species with the greatest changes in relative abundance before and after the intervention is shown in Figure 6. After the intervention, the relative abundances of *Bifidobacterium bifidum* and *Bifidobacterium kashiwanohense* PV2 significantly increased in the intestinal microbiota of children in the IG (*p* < 0.05), while no significant changes were observed in the CG (*p* > 0.05). In contrast, the abundance of *Bacteroides thetaiotaomicron* significantly increased in the CG (*p* < 0.05), but remained unchanged in the IG (*p* > 0.05). After the intervention, the abundance of *Bifidobacterium longum* was significantly higher in the IG compared to the CG (*p* < 0.05).

### 3.5 Effect of probiotic intervention on fecal immune parameters

#### 3.5.1 Effect of probiotic intervention on fecal pro-inflammatory/inflammatory factors

**Figure 7:**
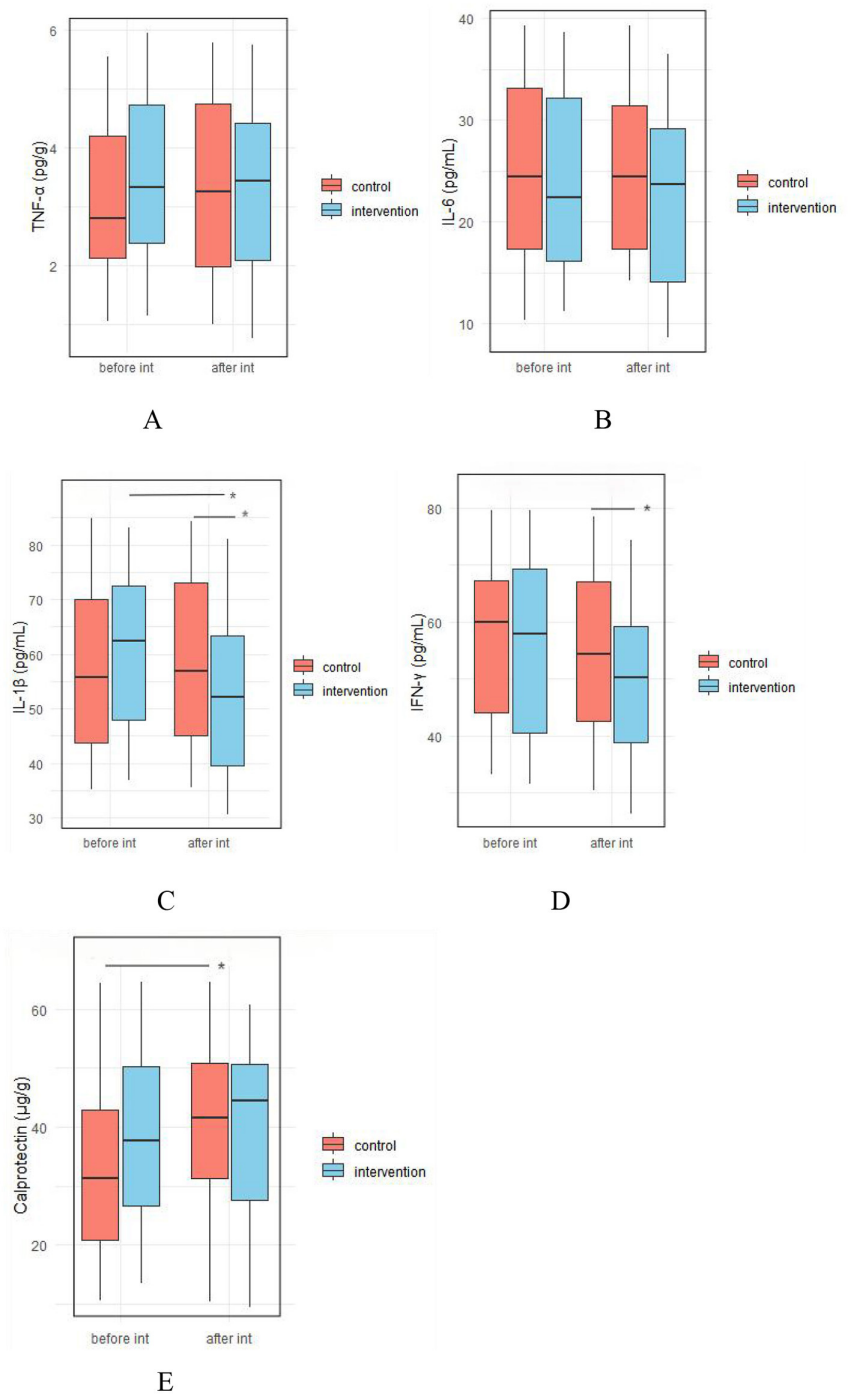
Effect of probiotic intervention on fecal pro-inflammatory/inflammatory factors. **(A)** TNF-α; **(B)** IL-6; **(C)** IL-1β; **(D)** IFNγ; **(E)**, calprotectin; control, placebo control group; intervention; probiotic intervention group; before int, before intervention; after int, after intervention. *, difference with significance (*p* < 0.05).

At baseline, there were no significant differences between the IG and the CG in fecal levels of TNF-α, IL-6, IL-1β, IFNγ, and calprotectin. After the intervention, the concentrations of IL-1β (51.98 ± 13.64 vs. 59.15 ± 15.74 pg/ml, *p* = 0.017) and IFNγ (49.76 ± 13.29 vs. 55.25 ± 14.02 pg/ml, respectively, *p* = 0.047) were significantly lower in the IG compared to the CG (Figure 7, Supplementary Table 3).

#### 3.5.2 Effect of probiotic intervention on fecal anti-inflammatory factors and immunoglobulin levels

**Figure 8:**
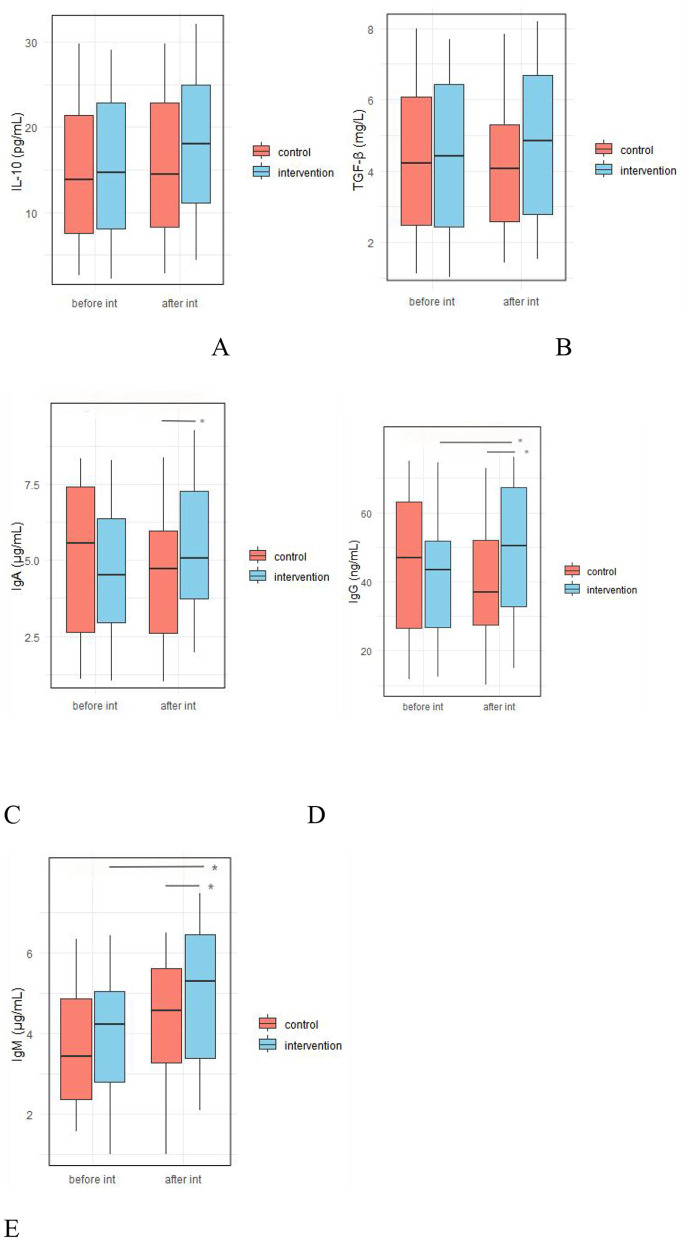
Effect of probiotic intervention on fecal anti-inflammatory factors and immunoglobulin levels. **(A)** IL-10; **(B)** TGF-β; **(C)** vIgA; **(D)** IgG; **(E)**, IgM; control, placebo control group; intervention, probiotic intervention group; before int, before intervention; after int, after intervention. *, difference with significance (*p* < 0.05).

As shown in Figure 8, there were no significant differences between the IG and the CG in baseline fecal levels of IL-10, TGF-β, IgA, IgG, and IgM. However, after the intervention, the IG shoed significantly higher levels of fecal IgA (5.39 ± 2.12 vs. 4.52 ± 2.17 μg/ml, *p* = 0.045), IgG (49.201 ± 18.93 vs. 39.53 ± 16.39 ng/ml, *p* = 0.007), and IgM (4.93 ± 1.68 vs. 4.18 ± 1.61 μg/ml, respectively, *p* = 0.026) compared to the IG (Figure 8, Supplementary Table 4).

#### 3.5.3 Effect of probiotic intervention on fecal allergy-related factors

**Figure 9:**
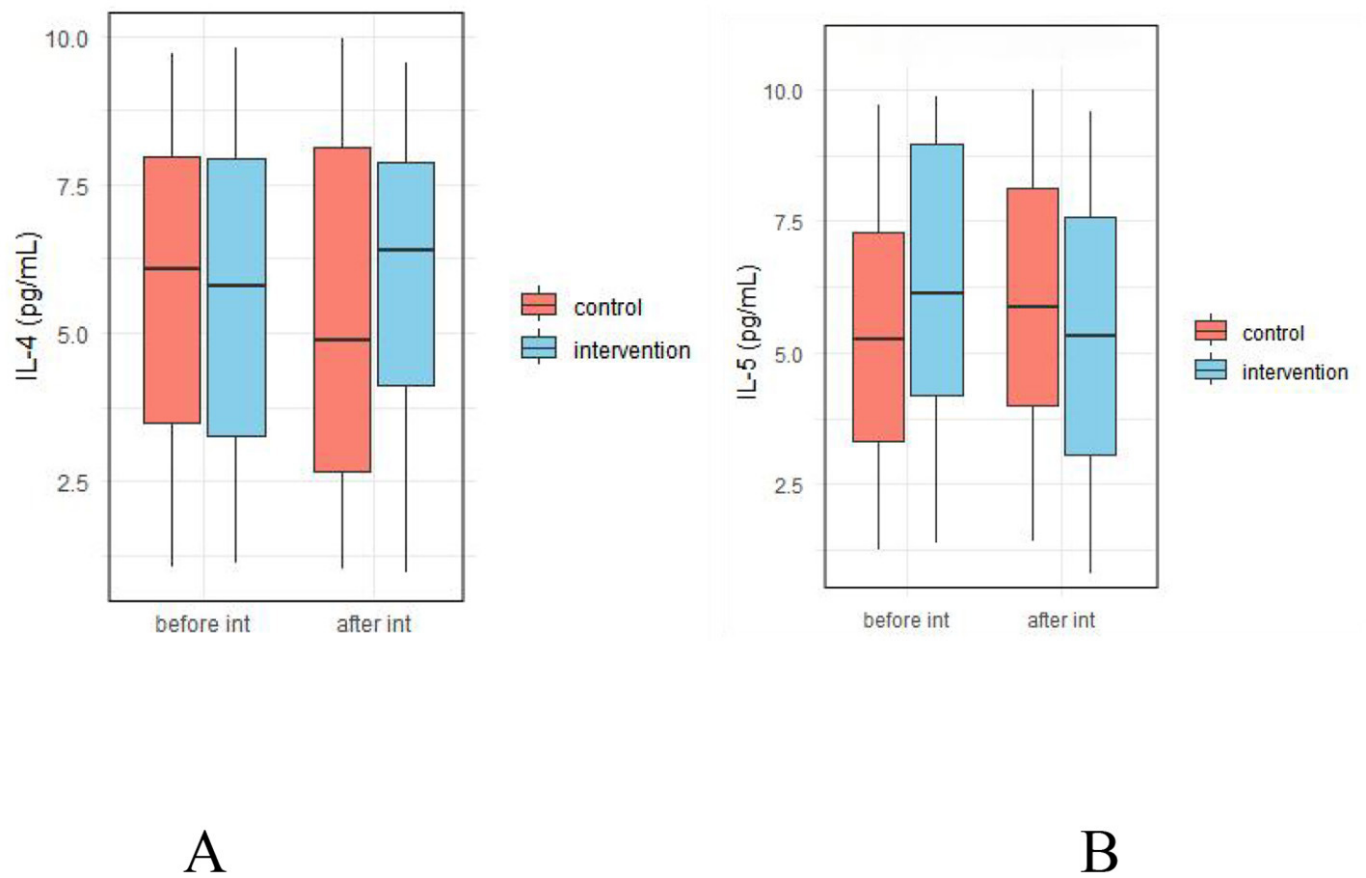
Effect of probiotic intervention on fecal allergy-related factors. **(A)** IL-4; **(B)** IL-5; control, placebo control group; intervention, probiotic intervention group; before int, before intervention; after int, after intervention.

As shown in Figure 9, there were no significant differences in fecal levels of IL-4 and IL-5 between the two groups before and after the intervention (*p* > 0.05, Supplementary Table 4).

#### 3.5.4 Effect of probiotic intervention on fecal SCFAs

**Figure 10:**
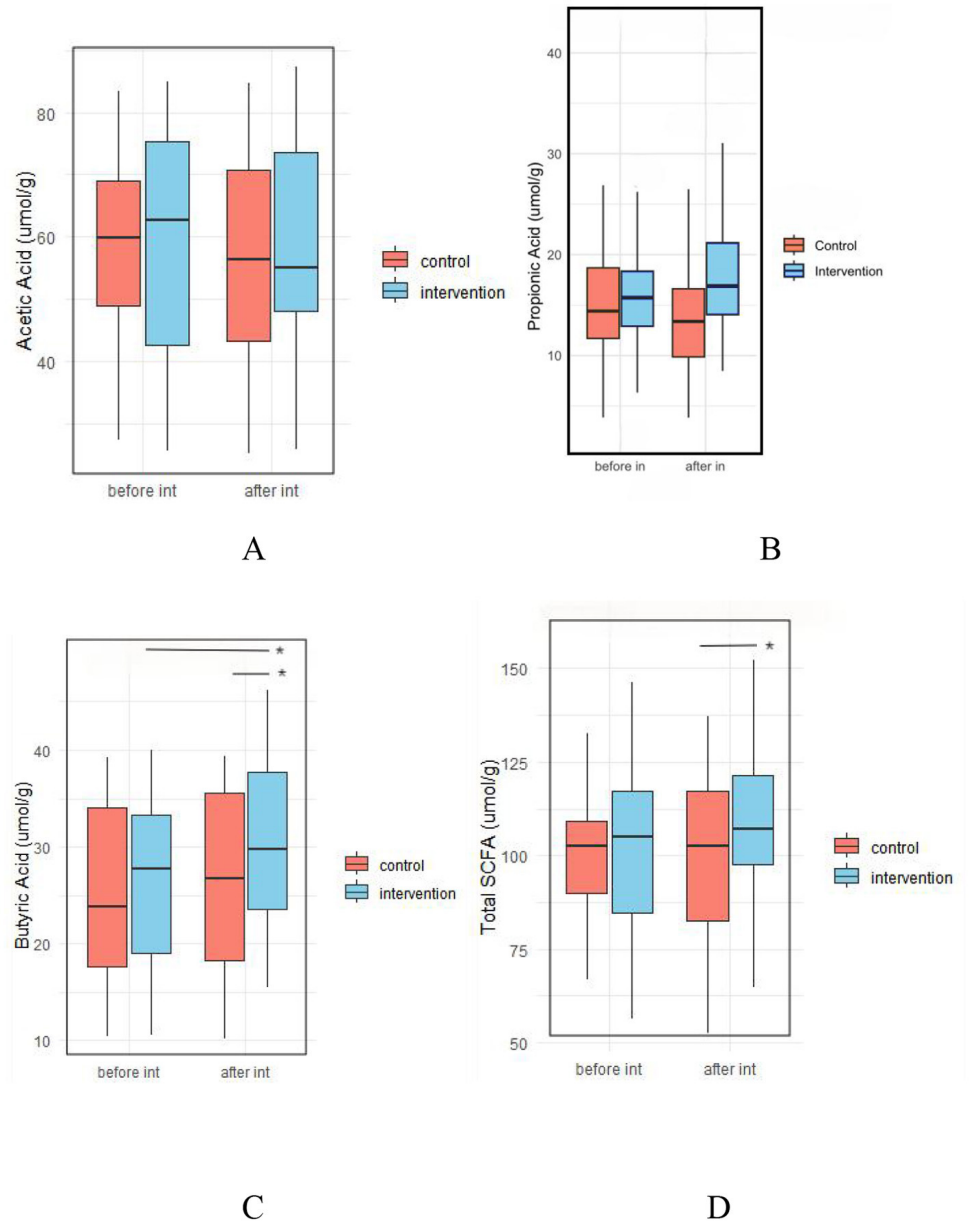
Effect of probiotic intervention on fecal SCFAs. **(A)** Acetic acid; **(B)** Propionic acid; **(C)** Butyric acid; **(D)** Total SCFA, total short chain fatty acids. control, placebo control group; intervention, probiotic intervention group; before int, before intervention; after int, after intervention. *, difference with significance (*p* < 0.05).

There were no significant differences in fecal levels of acetic acid, propionic acid, butyric acid, or total SCFAs between the two groups before the intervention (*p* > 0.05). However, after intervention, the levels of butyric acid (30.54 ± 8.92 vs. 26.32 ± 9.22 μmol/g, *p* = 0.022) and total SCFAs (108.97 ± 17.88 vs. 99.49 ± 22.18 μmol/g, *p* = 0.02) were significantly higher in the IG compared to the CG (Figure 10, Supplementary Table 4).

## 4 Discussion

### 4.1 Effect of probiotic intervention on clinical outcomes

There is growing support for using immunotherapy to strengthen the immune system and improve host defenses, particularly in early life, when immune function is still immature and less effective against infections (31). Probiotics have been shown to exert beneficial effects on the immune system, boost host defense mechanisms (32), restore balance in gut and respiratory microbiota, and reduce clinical manifestations of various diseases, including respiratory, gastrointestinal, and allergic diseases (13, 33–36).

The present study demonstrated that supplementation with YLGB-1496 significantly reduced the duration of cough, fever, dry stool, and eczematous changes of the skin. It also decreased the morbidity of URTI, bronchopneumonia, and eczema following a 3-month intervention, with effects observed over 6 months. These findings are largely consistent with previous studies.

Feedback from both caregivers and clinicians further supported these positive outcomes, highlighting the beneficial effects of YLGB-1496 on respiratory, gut, and immune health. Notably, no adverse effects were reported during the trial. Participants did not experience any probiotic-related side effects, such as abdominal cramps, nausea, vomiting, fever, diarrhea, constipation, appetite changes, or allergic reactions. Moreover, anthropometric measurements, including height, weight, and head circumference showed no significant differences between the IG and the CG. This confirms that the YLGB-1496 intervention improved respiratory, gut, and immune health without negatively affecting children's growth.

While our findings are consistent with the outcomes reported in many studies, it is important to acknowledge that probiotic efficacy is highly strain-specific and depends on individual strain characteristics (37, 38). Consequently, substantial heterogeneity exists across studies regarding the probiotic strain used, dosage administered, and duration of treatment. As a result, it remains unclear which specific probiotic strains, dosages and regimens are most effective for the prevention of respiratory, gastrointestinal, and allergic diseases.

### 4.2 Effect of probiotic intervention on fecal gut microbiota

In the current study, we explored the changes in gut microbiota composition and assessed the impact of probiotic supplementation on intestinal micro-ecology. These changes are closely linked to both the preventive effects and clinical progression of respiratory, gastrointestinal, and allergic diseases.

Intervention with *Bifidobacterium longum* has demonstrated extensive beneficial effects on the intestinal microecology of children, as confirmed by numerous clinical studies. For example, a recent study showed that a 3-month intervention with *Bifidobacterium lactis* Probio-M8 in children with asthma increased the abundance of potentially beneficial species such as *Bifidobacterium animalis, Bifidobacterium longum*, and *Prevotella* sp. Cytotoxin-Associated Gene (CAG), reduced *Parabacteroides distasonis* and *Clostridiales bacterium* and improved asthma symptoms (39). Similarly, Hiraku et al. (40) found that supplementation with *Bifidobacterium longum* M-63 promoted the development of *Bifidobacterium*-dominant gut microbiota during a critical developmental phase in term infants. In another study, *Bifidobacterium longum* BB536 was shown to alleviate upper respiratory illnesses in Malaysian pre-school children, accompanied by increased abundance of *Faecalibacterium* (41). Additional studies (42–45) have also demonstrated that various *Bifidobacterium longum* strains can alleviate disease symptoms and improve child health through their beneficial effects on gut microbiota. In the present study, intervention with YLGB-1496 significantly increased the relative abundances of *Bifidobacterium bifidum, Bifidobacterium kashiwanohense* and *Bifidobacterium longum*. These microbiota shifts were accompanied by improvements in common symptoms and low morbidity of URTIs, bronchopneumonia, and eczema. Although children in both groups experienced various respiratory, gastrointestinal, and allergic disorders during the follow-up period, the disease spectra and symptom durations were notably different.

These differences may be partially explained by specific changes in the gut microbiome. *Bifidobacterium* is well recognized as essential for the development of gut immunity and overall health in early childhood (46). As one of the initial microbes to inhabit the human gastrointestinal tract, *Bifidobacterium* plays a vital role in promoting immune function, protecting against pathogens, and modulating the immune system.

Interestingly, we observed a post-intervention increase in the relative abundance of *Bacteroides thetaiotaomicron* in the CG, while its levels remained stable in the IG. *B. thetaiotaomicron* is a prominent member of the human gut microflora and is known to influence host physiology by modulating gene expression related to mucosal barrier integrity, immune modulation, and nutrients metabolism (47, 48). Some studies have reported that interventions such as probiotics, nutritional supplements, food, and dietary changes can increase the relative abundance of *B. thetaiotaomicron* (49–51), while others found no significant changes (52–54). In some cases, this increase has been associated with improved health or recovery. At present, we do not have a definitive explanation for the increase in *B. thetaiotaomicron* observed in the CG. Several possible mechanisms may be involved: First, *B. thetaiotaomicron* has a strong capacity for regulating the intestinal microecological homeostasis. The immune and inflammatory status of children in the CG differed significantly from those in the IG, suggesting that the increase may be due to self-regulation of intestinal flora. Second, the relative increase could also be due to a decline in abundance of other beneficial bacterial species in the CG, resulting in a proportional rise in *B. thetaiotaomicron*. Future research is needed to confirm these findings.

### 4.3 Effect of probiotic intervention on fecal immune parameters

Overall, the present study showed that a 3-month intervention with YLGB-1496 led to a significant reduction in fecal anti-inflammatory factors (IL-1β and IFNγ), and an increase in immunoglobulins (IgA, IgG, and IgM) and SCFAs (butyric acid and total SCFAs). These immunological and metabolic improvements were accompanied by reduced episodes of common symptoms and lower risk ratios for URTIs, bronchopneumonia, and eczema. Although the YLGB-1496 intervention had little effect on allergy-related factors, it still significantly reduced the risk of eczema in children.

Numerous studies have confirmed that *Bifidobacterium longum* can influence various components of the human intestinal immune system, and these effects are closely associated with clinical manifestations and health status. However, the specific immune-modulating effects vary significantly depending on the strain, host characteristics, disease type, and mode of intervention (55–60). This study only showed that the YLGB-1496 intervention reduced the episodes of four symptoms and three diseases, its effect on other common conditions appeared limited. However, our data revealed significant differences in intestinal immunity, inflammatory status, SCFA levels, and microbial metabolites between the IG and the CG. The absence of broader protective effects may be attributed to limitations such as insufficient sample size, relatively short observation period, and the limited sensitivity of clinical indicators used.

SCFAs, including butyrate, propionate, and acetate, are microbial metabolites whose abundance are shaped by environmental factors such as diet and the use of antibiotics and probiotics (61). SCFAs play essential roles in regulating epithelial barrier function, mucosal immunity, and systemic immune responses (62). Several studies have also reported that *Bifidobacterium longum* can modulate SCFA production, with these changes correlating with clinical improvement (40, 63–65). In this study, children in the IG showed significantly higher fecal levels of butyric acid and total SCFAs after supplementation with YLGB-1496 compared to the CG. These findings were consistent with the observed increases in fecal immunoglobulins, changes in cytokines, and improved clinical manifestations, suggesting that the beneficial effects of the probiotic are closely related to increased SCFA production.

### 4.4 Limitation analysis

Firstly, the sample size of this study is relatively small. As a result, the statistical power may have been insufficient to detect significant effects for diseases with lower incidence rates. Secondly, the study used a single dose of YLGB-1496 (1.5 × 10^10^ CFU/day), which limited our ability to explore the dose–response relationship and determine the optimal dosage for preventing respiratory, gastrointestinal, and allergic diseases.

Finally, the impact of exogenous probiotic supplementation on intestinal function may be affected by various confounding factors, including mode of delivery, feeding method, dietary patterns, and environmental conditions. While some of these factors were considered in the analysis, their potential impact may have attenuated the observed effects of YLGB-1496. Future research should consider increasing the sample size and prolonging the observation period to enhance the reliability and representativeness of the findings. Further investigation into the long-term health effects and optimal dosing strategies for YLGB-1496 in pediatric populations is also warranted.

## 5 Conclusion

In conclusion, our study did not observe any adverse effects associated with the YLGB-1496 intervention, indicating its safety for use in healthy children. Daily supplementation with YLGB-1496 at a dosage of 1.5 × 10^10^ CFU for 3 months effectively reduced the duration of cough, fever, dry stool, and eczematous changes of the skin, and significantly decreased the morbidity of URTI, bronchopneumonia, and eczema in children. The intervention also positively modulated gut microbiome composition and enhanced immune function without any adverse effects.

## Data Availability

The raw 16S rRNA gene sequences for all fecal samples used in this study have been deposited in the National Center for Biotechnology Information BioProject database under the BioProject ID PRJNA1231027.
