# Correction: Effect of *Bifidobacterium longum* subsp. *infantis* YLGB-1496 on common diseases in pediatrics: a randomized, blinded, placebo-controlled trial

**DOI:** 10.3389/fnut.2025.1661669

**Published:** 2025-08-04

**Authors:** Xi Zhang, Ke Chen, Hanglian Lan, Haixia Chen, Hua Chen, Ping Yang, Nianyang He, Weilian Hung, Zaozhong Zeng, Changqi Liu

**Affiliations:** ^1^Chengdu Women's and Children's Central Hospital, School of Medicine, University of Electronic Science and Technology of China, Chengdu, China; ^2^Department of Nutrition, Chengdu Women's and Children's Central Hospital, School of Medicine, University of Electronic Science and Technology of China, Chengdu, China; ^3^National Center of Technology Innovation for Dairy, Hohhot, China; ^4^Baoxing Center for Disease Control and Prevention, Yaan, China; ^5^Department of Child Health Care, Xindu Maternal and Child Health Care Hospital, Chengdu, China; ^6^School of Exercise and Nutritional Sciences, San Diego State University, San Diego, CA, United States

**Keywords:** probiotics, short chain fatty acids, children, gut microbiota, upper respiratory tract infection

In the published article, there was an error in Table 2, “Comparison of the days of common symptoms in infants and young children during the intervention period” as published.

The positions of “IG” and “CG” in the column headings were erroneously swapped. Similarly, “44 0.89” and “99 2.2” in the “Fever” row were incorrectly switched.

The corrected Table 2 appears below.

**Table 2:** Comparison of the days of common symptoms in infants and young children during the intervention period.

**Common symptoms**	**CG (*****N*** = **50)**		**IG (*****N*** = **50)**		***p-*value**
	**Total occurrence days**	**Incident rate per 100 intervention days (%)^†^**	**Total occurrence days**	**Incident rate per 100 intervention days (%)^†^**	
Cough^*^	190	4.22	99	2.2	< 0.001
Runny nose	171	3.8	136	3.02	0.133
Stuffy nose	120	2.67	83	1.84	0.066
Fever^*^	99	2.2	40	0.89	< 0.001
Loose stools	97	2.16	120	2.67	0.413
Increased frequency of bowel movements	105	2.33	105	2.33	0.921
Colic of the intestines	105	2.33	102	2.27	0.979
Dry stool^*^	346	7.69	157	3.49	0.012
Choking	45	1	58	1.29	0.387
Increased belching/bloating/anal gas	87	1.93	89	1.98	0.915
Retching/vomiting	35	0.78	52	1.16	0.244
Stool with milk flaps/food residues/sour odor	183	4.07	194	4.31	0.867
Reflux	58	1.29	64	1.42	0.393
Decreased appetite	181	4.02	135	3	0.285
Dysphoria	225	5	151	3.36	0.659
Refusal to eat	83	1.84	145	3.22	0.065
Eczematous changes of the skin^*^	578	12.84	134	2.98	0.002
Erythematous changes in the skin	12	0.27	9	0.2	0.958
Skin wheal-like changes	10	0.22	7	0.16	0.552

^†^A total of 9,000 study days (calculated method: 180 days × 50 people). Symptom incidence per 100 intervention days = duration of a symptom/total intervention days × 100%.

^*^Mann–Whitney *U*-test for two independent samples, *p*-values < 0.01. Dry stool refers to stool Bristol score type 1–3 stool.

IG, intervention group; CG, control group.

The original article has been updated.

