# Supplementary material for: Effect of Bifidobacterium longum subsp. infantis YLGB-1496 on common diseases in pediatrics: a randomized, blinded, placebo-controlled trial
Source: Front Nutr. 2025 Jun 27;12:1585504. doi: 10.3389/fnut.2025.1585504 (PMC12261461; doi:10.3389/fnut.2025.1585504)
Supplement: Supplementary file 1 [file Supplementary_file_1.docx]

Table 1 Negative binomial regression analysis of common symptoms and days of occurrence in infants and young children during the study period was compared between groups

| **Main Symptom** | **Intervention Method** | **Parameter Estimate (B)** | **Standard Error** | **95% Wald Confidence Interval** | | **Hypothesis Test** | | |
| --- | --- | --- | --- | --- | --- | --- | --- | --- |
|  |  |  |  | Lower Bound | Upper Bound | Wald Chi-square | df | Sig. |
| Cough | Placebo Control Group | 0.549 | 0.2842 | -0.008 | 1.106 | 3.736 | 1 | 0.053 |
|  | Probiotic Supplement Group | 0a | . | . | . | . | . | . |
| Runny Nose | Placebo Control Group | 0.235 | 0.2747 | -0.303 | 0.773 | 0.731 | 1 | 0.393 |
|  | Probiotic Supplement Group | 0a | . | . | . | . | . | . |
| Nasal Congestion | Placebo Control Group | 0.252 | 0.2819 | -0.301 | 0.804 | 0.799 | 1 | 0.371 |
|  | Probiotic Supplement Group | 0a | . | . | . | . | . | . |
| Fever | Placebo Control Group | 1.064 | 0.3274 | 0.422 | 1.705 | 10.553 | 1 | 0.001 |
|  | Probiotic Supplement Group | 0a | . | . | . | . | . | . |
| Loose Stool | Placebo Control Group | -0.113 | 0.2995 | -0.700 | 0.474 | 0.142 | 1 | 0.707 |
|  | Probiotic Supplement Group | 0a | . | . | . | . | . | . |
| Increased Stool Frequency | Placebo Control Group | 0.024 | 0.2803 | -0.526 | 0.573 | 0.007 | 1 | 0.932 |
|  | Probiotic Supplement Group | 0a | . | . | . | . | . | . |
| Colic | Placebo Control Group | -0.100 | 0.2884 | -0.665 | 0.465 | 0.120 | 1 | 0.729 |
|  | Probiotic Supplement Group | 0a | . | . | . | . | . | . |
| Constipation | Placebo Control Group | 0.863 | 0.2676 | 0.339 | 1.387 | 10.402 | 1 | 0.001 |
|  | Probiotic Supplement Group | 0a | . | . | . | . | . | . |
| Choking on Milk | Placebo Control Group | -0.352 | 0.3405 | -1.019 | 0.316 | 1.068 | 1 | 0.302 |
|  | Probiotic Supplement Group | 0a | . | . | . | . | . | . |
| Excessive Gas/Flatulence | Placebo Control Group | 0.144 | 0.3047 | -0.453 | 0.742 | 0.224 | 1 | 0.636 |
|  | Probiotic Supplement Group | 0a | . | . | . | . | . | . |
| Dry Heaving/Vomiting | Placebo Control Group | -0.506 | 0.3398 | -1.172 | 0.160 | 2.220 | 1 | 0.136 |
|  | Probiotic Supplement Group | 0a | . | . | . | . | . | . |
| Milk Curds/Food Residues (Sour Odor) | Placebo Control Group | 0.067 | 0.2624 | -0.448 | 0.581 | 0.064 | 1 | 0.800 |
|  | Probiotic Supplement Group | 0a | . | . | . | . | . | . |
| Reflux | Placebo Control Group | -0.093 | 0.3339 | -0.747 | 0.562 | 0.077 | 1 | 0.781 |
|  | Probiotic Supplement Group | 0a | . | . | . | . | . | . |
| Decreased Appetite | Placebo Control Group | 0.145 | 0.2715 | -0.387 | 0.677 | 0.284 | 1 | 0.594 |
|  | Probiotic Supplement Group | 0a | . | . | . | . | . | . |
| Irritability | Placebo Control Group | 0.326 | 0.2621 | -0.187 | 0.840 | 1.551 | 1 | 0.213 |
|  | Probiotic Supplement Group | 0a | . | . | . | . | . | . |
| Food Refusal | Placebo Control Group | -0.371 | 0.2727 | -0.905 | 0.163 | 1.851 | 1 | 0.174 |
|  | Probiotic Supplement Group | 0a | . | . | . | . | . | . |
| Eczema-like Rash | Placebo Control Group | 1.508 | 0.2520 | 1.014 | 2.002 | 35.823 | 1 | <0.001 |
|  | Probiotic Supplement Group | 0a | . | . | . | . | . | . |

Note:

1. "0a" indicates the reference category in statistical analysis
2. "." represents missing/unavailable data
3. The original Chinese decimal separator "." is preserved
4. Statistical significance levels are maintained (e.g., <0.001)

Table 2 Effect of probiotic intervention on the growth and development of infants and young children (mean ± sd)

| index | | intervention group (N=50) | control group (N=50) | *t* values | *p* values |
| --- | --- | --- | --- | --- | --- |
| **height (cm)** | Pre-intervention* | 75.4±12.8 | 78.5±12.3 | 1.235 | 0.218 |
|  | After-intervention* | 79.6±14.7 | 82.6±11.0 | 1.155 | 0.251 |
| **weight(kg)** | Pre-intervention * | 10.3±3.4 | 10.1±3.0 | 0.312 | 0.756 |
|  | After-intervention * | 13.1±4.4 | 11.8±3.0 | 1.726 | 0.087 |
| **Head circumference(cm)** | Pre-Intervention* | 44.5±4.0 | 45.3±3.5 | 1.064 | 0.289 |
|  | After-intervention* | 47.5±4.4 | 47.0±2.8 | 0.678 | 0.502 |

Table 3 Effects of intervention on fecal immunity and fatty acid-related biochemical indexes in infants and young children (mean ± standard deviation)

| Fecal biochemical indicators | |  | control group (N=50) | intervention group (N=50) | *t* values | *p* values |
| --- | --- | --- | --- | --- | --- | --- |
| **Proinflammatory/inflammatory factors** | TNF-α(µg/L) | Pre-intervention | 3.13±1.29 | 3.48±1.38 | 1.289 | 0.201 |
|  |  | Post-intervention | 3.33±1.55 | 3.23±1.52 | 0.306 | 0.760 |
|  | IL-6(pg/mL) | Pre-intervention | 25.12±8.66 | 24.00±8.67 | 0.642 | 0.522 |
|  |  | Post-intervention | 24.46±7.52 | 22.90±8.58 | 0.970 | 0.334 |
|  | IL-1β(pg/mL) | Pre-intervention | 57.79± 15.54 | 60.89± 13.47 | -1.064 | 0.29 |
|  |  | Post-intervention | 59.15± 15.74 | 51.98 ±13.64 | 2.432 | 0.017 |
|  | IFNγ(pg/mL) | Pre-intervention | 57.03± 13.96 | 54.94 ±15.79 | 0.699 | 0.48 |
|  |  | Post-intervention | 55.25± 14.02 | 49.76±13.29 | 2.007 | 0.047 |
|  | Calprotectin (µg/g) | Pre-intervention | 33.17±14.55 | 38.56± 15.09 | -1.817 | 0.07 |
|  |  | Post-intervention | 40.09± 15.55 | 39.36± 15.09 | 0.239 | 0.811 |
| **Anti-inflammatory factors** | IL-10(pg/mL) | Pre-intervention | 15.004 ± 8.15 | 15.28 ±8.48 | -0.168 | 0.867 |
|  |  | Post-intervention | 15.53±8.33 | 18.33±8.38 | -1.679 | 0.096 |
|  | TGF-β(mg/L) | Pre-intervention | 4.29 ± 2.16 | 4.45 ± 2.15 | -0.354 | 0.723 |
|  |  | Post-intervention | 4.16 ± 1.79 | 4.75± 2.14 | -1.493 | 0.139 |
|  | IgA(ug/mL) | Pre-intervention | 5.09 ±2.45 | 4.58 ±2.22 | 1.09 | 0.278 |
|  |  | Post-intervention | 4.52±2.17 | 5.39±2.12 | -2.025 | 0.045 |
|  | IgG(ng/mL) | Pre-intervention | 45.55±19.102 | 41.65± 17.44 | 1.067 | 0.288 |
|  |  | Post-intervention | 39.53±16.39 | 49.201 ±18.93 | -2.729 | 0.007 |
|  | IgM(ug/mL) | Pre-intervention | 3.6602 ± 1.41 | 3.9640 ±1.49 | -1.046 | 0.298 |
|  |  | Post-intervention | 4.18 ± 1.61 | 4.93 ±1.68 | -2.255 | 0.026 |
| **Allergy-related factors** | IL-4(pg/mL) | Pre-intervention | 5.706 ±2.54 | 5.64 ± 2.68 | 0.112 | 0.911 |
|  |  | Post-intervention | 5.301±2.94 | 5.88 ± 2.43 | -1.07 | 0.286 |
|  | IL-5(pg/mL) | Pre-intervention | 5.29± 2.46 | 6.31 ± 2.61 | -2.021 | 0.051 |
|  |  | Post-intervention | 5.79 ± 2.45 | 5.35±2.59 | 0.868 | 0.387 |
| **Short-chain fatty acids** | acetic acid (umol/g) | Pre-intervention | 58.63± 13.80 | 58.74 ±18.82 | -0.033 | 0.973 |
|  |  | Post-intervention | 56.21±18.37 | 58.83 ±16.09 | -0.759 | 0.449 |
|  | propionic acid (umol/g) | Pre-intervention | 14.806 ± 5.563 | 14.89 ±5.53 | -0.077 | 0.938 |
|  |  | Post-intervention | 15.04±5.45 | 17.42±6.83 | -1.927 | 0.0569 |
|  | butyric acid (umol/g) | Pre-intervention | 25.25±9.24 | 26.34±8.66 | -0.604 | 0.547 |
|  |  | Post-intervention | 26.32±9.22 | 30.54±8.92 | -2.326 | 0.022 |
|  | Total short-chain fatty acids (umol/g) | Pre-intervention | 100.67±16.708 | 102.05±21.21 | -0.362 | 0.718 |
|  |  | Post-intervention | 99.49± 22.18 | 108.97±17.88 | -2.352 | 0.02 |


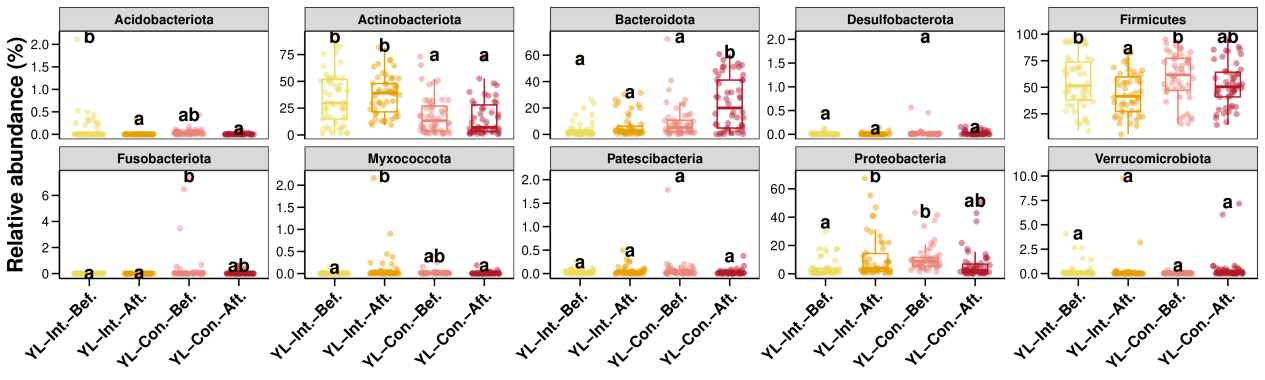


A


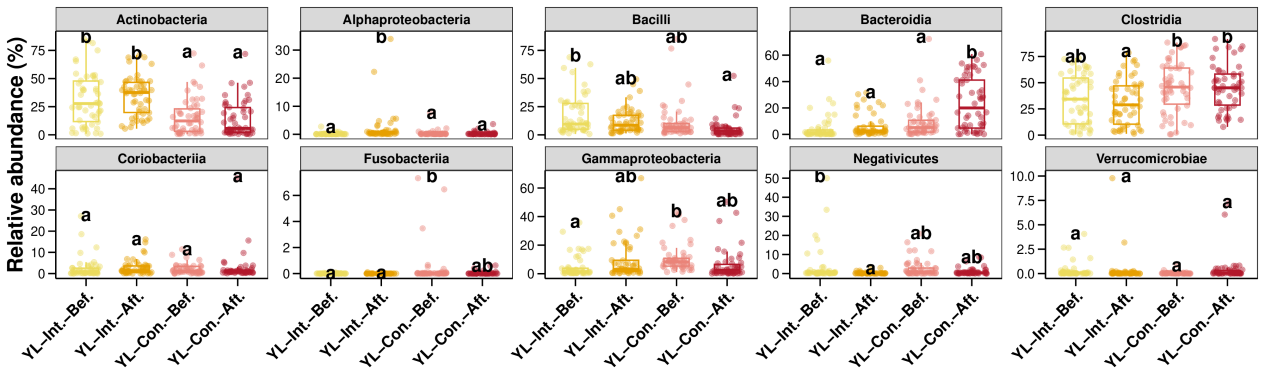


B


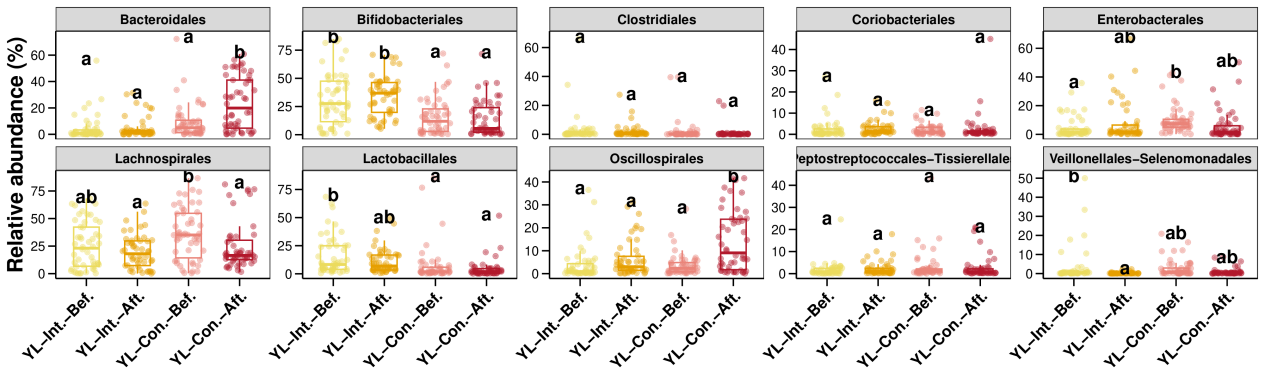


C


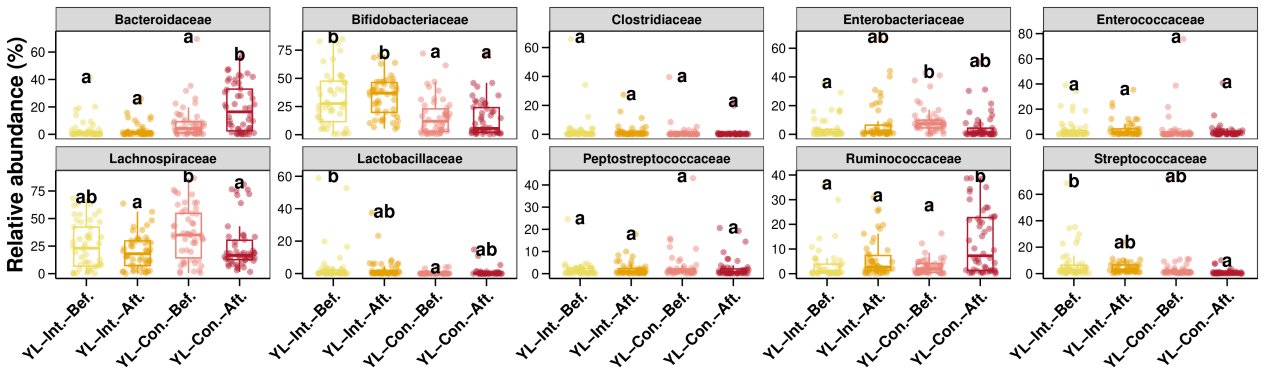


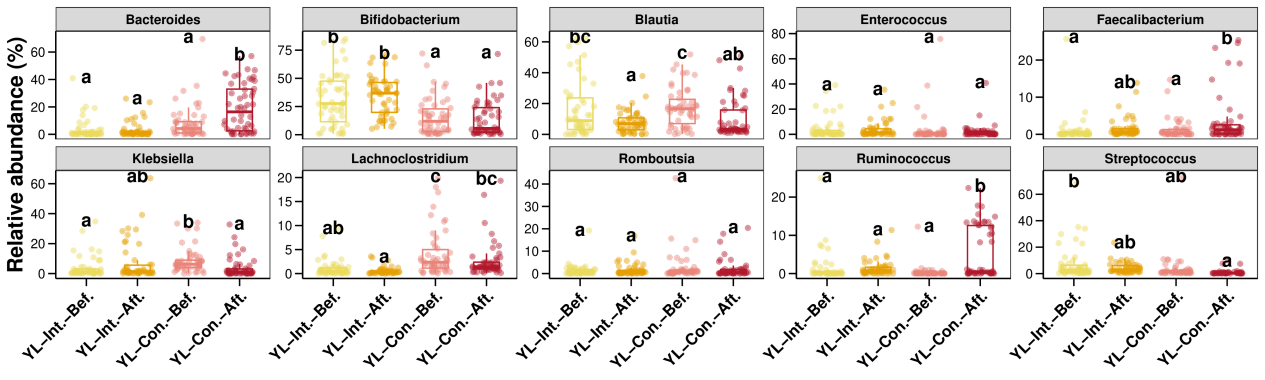


D


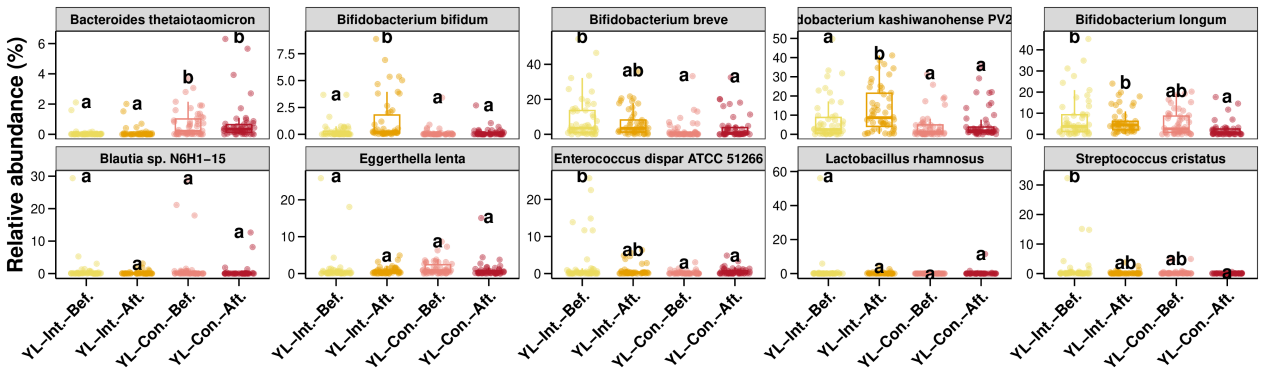


E

FigS Comparison of the top 10 differences in relative abundance between groups before and after intervention

A, phylum level; B, class level; C, order level; D, family level; E, genus level; Bla-Pre-treat, Probiotic IG before intervention; Bla-1-treat, After the intervention in the probiotic IG; Con-Pre-treat, Placebo CG before intervention; Con-1-treat, After the intervention in the placebo CG.ab: There was no significant difference between groups with the same letters (*p*>0.05); the difference between groups with different letters was statistically significant (*p*<0.05).
